# pH-Dependent Adsorption of Human Serum Albumin Protein on a Polystyrene-Block–Poly(acrylic acid)-Coated PVDF Membrane

**DOI:** 10.3390/membranes13120886

**Published:** 2023-11-22

**Authors:** Charaf-Eddine Merzougui, Pierre Aimar, Patrice Bacchin, Christel Causserand

**Affiliations:** Laboratoire de Génie Chimique, Université de Toulouse, CNRS, INP, UPS, 31062 Toulouse, France; pierre.aimar@univ-toulouse.fr (P.A.); patrice.bacchin@univ-tlse3.fr (P.B.)

**Keywords:** PVDF membrane, PS-b-PAA copolymer, coating, HSA adsorption, pH dependence

## Abstract

This study reports the investigation of human serum albumin (HSA) adsorption on a poy-styrene-block–poly(acrylic acid) (PS-b-PAA)-coated PVDF membrane, which is a potential smart material for biomedical applications. First, copolymer coating on the membrane surface was successfully performed, due to the hydrophobic interaction of the PS anchoring group with the PVDF membrane. This was confirmed by Fourier transform infrared spectroscopy (FTIR) characterization of the membrane. Then, HSA adsorption onto the coated membrane was assessed and was proved to be strongly dependent on the pH of the protein solution. Indeed, both FTIR mapping and mass balance calculation using UV–visible spectroscopy displayed a greater HSA adsorption on the membrane at pH 5, even though it still took place at higher pH, but to a lower extent. Afterwards, an ionic strength influence study evinced the role of electrostatic interactions between HSA and the PAA layer on HSA adsorption. Dead-end filtration of HSA through the coated membrane confirmed the pH dependence of HSA adsorption on the coated membrane.

## 1. Introduction

The interactions of proteins with polyelectrolytes have been a major topic of interest in colloids and interface science for a few decades [1,2,3,4]. They are involved in several phenomena in chemistry and biology [5,6] and in various applications such as drug delivery [7,8,9], protein purification [10] or extraction [11], enzyme stabilization [12,13], and medical applications [14]. In many of these applications, the polyelectrolytes are in the form of charged polymer chains end-attached to an interface to promote the adsorption of specific proteins depending on the application. On the other hand, the nonspecific adsorption of proteins on these interfaces is to be prevented to avoid fouling. For instance, in biomedical and biotechnological applications, artificial medical interfaces such as implants are exposed to biological liquids [15]. This could induce the nonspecific adsorption of proteins, leading to the formation of biofilms and so, to biofouling [14]. Wherefore, many studies were previously conducted to understand proteins–polyelectrolytes interactions in bio-systems [16,17]. Thus, the understanding and the control of these interactions is required because they are the key factors for the efficiency of potential applications.

Commonly, plasma proteins such as Bovine Serum Albumin (BSA) or Human Serum Albumin (HSA) are the most used [18,19] for the study of the interactions between biological proteins and charged interfaces. In fact, the adsorption of serum albumin on coated surfaces in biomedical applications is widely studied to gain insights in the fouling of the surfaces [19], because albumin is the most abundant protein in plasma [20].

As for polyelectrolytes, poly acrylic acid (PAA) is a negatively charged polymer that has been extensively used for the investigation of proteins–polyelectrolytes interactions. In fact, PAA and its derivatives are widely used as materials in biomedical-related applications such as drug delivery [21,22], due to their biocompatibility, highlighted in various studies [23].

Hence, many articles reported the study of the interactions of proteins with planar PAA brushes [24,25,26,27] or with PAA-grafted interfaces, mostly nanoparticles [14,28,29,30]. Moreover, most of these studies discussed the interaction of PAA with BSA or HSA. Accordingly, BSA was shown to adsorb on planar PAA brushes using optical reflectometry, with maximum adsorption observed near the isoelectric point (IP ≈ 4.7) of the protein [24]. This was attributed to a phenomenon called charge regulation that concerns the proton equilibria of some sites on the protein, which are perturbed when approached by other proteins, membranes, polyelectrolytes, etc. [31]. The adsorption was then disclosed to remain substantial above the IP and was proved to be dependent on the ionic strength, since it decreased with the increase in salt concentration. The same behavior was observed for HSA, whose binding with small polyelectrolytes PAA brushes was examined using isothermal calorimetry (ITC) [27]. In fact, at pH 7.2, a study on salt concentration effect revealed that this binding was mainly due to the attractive electrostatic potential between the two solutes.

The adsorption of BSA on PAA-grafted spherical polyelectrolyte brushes was also previously studied at pH 6.1, and it was found that BSA adsorbed strongly at low salt concentrations and that this adsorption decreased drastically with the increase in the ionic strength [28]. Eventually, all these studies evinced the ionic strength-dependent adsorption of serum albumin proteins on PAA polyelectrolytes at pH values different from the IP, at which it is theoretically supposed not to take place. Indeed, at pH values above the IP, both protein and polyelectrolyte are negatively charged, since PAA possesses a pKa of 4.5. Thus, the protein–polyelectrolyte interaction was attributed to either the release of counter-ions and the presence of positive patches on the protein surface [18] or the charge regulation of the protein [19,24]. Yet, it was established that the adsorption strength depended on the pH, though it still happened even above pH 5. On the other hand, the formation of complexes between HSA and 100 kDa PAA brushes was demonstrated in our previous work using small-angle X-ray scattering (SAXS) [32], but only at a pH close to 5. This specific condition may be necessary to satisfy two requirements: (i) the reduction of the overall negative charge of the protein in order to lower electrostatic repulsions between HSA and PAA, (ii) the persistence of electrostatic attractions between the negatively charged PAA chains and the positive patches on the protein surface in order to form HSA–PAA complexes.

Other studies extended the investigation of proteins–polyelectrolytes interactions to the interface of grafted membranes [33,34,35,36,37]. In fact, their aim was either to reduce biofouling and enhance the membrane biocompatibility [38,39,40] or to develop smart membranes for biomedical applications such as blood separation [23,41]. Among these studies, few discussed the effect of the physicochemical parameters such as pH and ionic strength on protein adsorption on grafted membranes, previously shown to be the decisive factors controlling adsorption, as stated above.

In the present study, the adsorption of HSA on PVDF membranes coated with polystyrene-block–poly(acrylic acid) (PS-b-PAA) copolymers was explicitly explored using Fourier transform infrared spectroscopy (FTIR). In fact, the static adsorption of HSA on PVDF membranes coated with PS-b-PAA copolymers of different sizes was first tested at constant pH and ionic strength. The amount of absorbed HSA was evaluated by mass balance using UV–visible spectroscopy. Afterwards, both pH and ionic strength were varied to investigate their influence on HSA adsorption onto the PS-b-PAA-coated membranes. Furthermore, the membrane transport properties were assessed by measuring the permeability of coated and pristine membranes. Then, filtration of the HSA solutions was performed at neutral pH, and HSA adsorption onto the membrane was explored. Eventually, the pH effect on HSA adsorption onto the coated membranes was evinced during filtration.

## 2. Materials and Methods

### 2.1. PS-b-PAA Solution Preparation

The polystyrene-b–poly(acrylic acid) diblock copolymers used in this study (listed in Table 1) were provided by Sigma-Aldrich, Burlington, MA, USA, (PS_30_-b-PAA_5_) and by Polymer Source, Inc., (Dorval, QC, Canada, PS_26_-b-PAA_76_ and PS_100_-b-PAA_107_). The copolymers were dissolved in a 50% (*v*/*v*) solvent consisting of absolute ethanol (EtOH_abs_) and tetrahydrofuran (THF), which were, respectively, purchased from VWR Chemicals Avantor^®^ (Radnor, PA, USA) and Acros Organics (Geel, Belgium). They were dissolved at room temperature (around 22 ± 3 °C) to obtain a PS-b–PAA stock solution of 10 mg·mL^−1^, from which samples at lower concentrations were prepared by dilution. The samples were placed in a ultrasonic bath for 10 min to ensure the complete dissolution of the copolymer.

### 2.2. Coating of PVDF Membranes

Commercial polyvinylidene fluoride (PVDF) microporous hydrophobic membranes (VVHP, Millipore Co., Burlington, MA, USA) with a nominal average pore size of 0.1 μm and a thickness of 125 μm were used as received. For coating, an area of 0.5 cm^2^ of the PVDF membrane was immersed for 2 h in 1 mL of the copolymer solution at the desired concentration. The membrane was then drained and left exposed to the air for a while before it was placed in a Petri dish in an oven to dry at 40 °C for 2 h for further characterization. All experiments were conducted at a constant copolymer concentration that was set at 5 mg·mL^−1^. It is important to note that the experiments were performed at room temperature, at around 22 ± 3 °C.

### 2.3. Static Adsorption of Blood Proteins

A blood protein was used for the protein adsorption study; albumin from human serum (HSA, A1653) was purchased from Sigma-Aldrich, Burlington, MA, USA. The protein solutions were prepared by dissolving 1 mg of HSA in 1 mL of PBS 1X (BP399, Fisher BioReagents, Pittsburgh, PA, USA, pH = 7.4; I = 137 mM). They were prepared the day before the adsorption experiment and kept refrigerated. The membrane was coated as explained previously and was then immersed in 1 mL of PBS 1X overnight to hydrate the copolymer structure. Afterwards, PBS was replaced with 1 mg·mL^−1^ of protein solution for 2 h at room temperature. Then, the membrane was rinsed three times with PBS to remove the non-adsorbed proteins and finally was dried in the oven for 2 h at 35 °C for further characterization.

For the experiments in which the protein solutions used for the immersion of the coated membranes had different pH values, the pH was controlled using different buffer solutions at 0.1 M (TRIS base pH 8, HEPES pH 7, and a mixture of sodium citrate di-hydrate and citric acid at pH 5). In the meantime, the ionic strength of these protein solutions was fixed by adding the required amounts of a 4 M NaCl solution.

### 2.4. ATR-FTIR for Membrane Surface Characterization

ATR-FTIR was shown to be effective in the study of protein adsorption onto membranes [42]. Thus, FTIR mapping was used in this study due to its ease of operation and simplicity. The membrane surfaces were scanned with an infrared spectrometer (IN10MX Thermo Scientific, Waltham, MA, USA) under ATR mode using a germanium crystal with a 25° incident angle. The spectrometer was equipped with an MCT-A detector cooled with liquid nitrogen and a KBr beam splitter. The spectral resolution was 8 cm^−1^, and 16 scans were acquired on each measurement point. Then, 50 × 50 points were measured (with a step size of 100 µm between 2 points), covering a total area of 25 mm^2^ that was used for surface chemical mapping to determine the coverage of the coating and the protein adsorption.

The files obtained from the FTIR mapping were first processed with the OMNIC Software Suite (OMNIC Atlµs v.9.2, Thermo Fisher Scientific), which allowed the scanned maps to be drawn.

To generate chemical maps displaying the qualitative distribution of the copolymer or the protein over the membrane surface, the peaks of interest representing their chemical signature and confirming their presence were first identified and are summarized in Supplementary Materials Table S1. For that, Attenuated Total Reflectance (ATR) spectroscopy (Nicolet 6700, Thermo Scientific) with a diamond crystal, a 45° incident angle, 16 scans, and a 4 cm^−1^ spectral resolution was used to obtain spectra of pure copolymers and protein (Supplementary Materials Figure S1). Peaks with the highest absorbance and unique to the copolymer or the protein were chosen for chemical mapping. Then, their areas were measured (Supplementary Materials Figure S2) to generate the chemical maps, which were color-coded according to absorption peak intensity, from blue (lowest intensity) to red (highest intensity). A higher peak intensity indicated more extensive coating or protein presence on the surface. All maps generated were unprocessed, except for atmospheric and the baseline corrections. For all experiments, the copolymer distribution was analyzed using the peak at 700 cm^−1^, while that of the protein was assessed using the peak at 1660 cm^−1^.

It is important to note that the experiments of membrane coating with the copolymer and of protein adsorption on the membrane, as well as the mapping, were repeated from 2 to 3 times to confirm the reproducibility of the results.

### 2.5. UV–Visible Spectroscopy for Protein Adsorption Analysis

UV–visible spectroscopy was used to evaluate the amount of protein adsorbed onto the membrane surface by analyzing the solute concentration before and after immersing the membrane in the solution. The quantity of the adsorbed protein was then calculated by mass balance using the equation:

(1) $$m_{ads}=C_{i}\times V-C_{eq}\times V\;$$

where *m_ads_* is the adsorbed mass of the protein, *C_i_* and *C_eq_* are the initial concentration and that at equilibrium, respectively of the concerned solute (protein), and *V* is the volume.

It is important to note that even if the initial concentrations were already known, the concentrations of the copolymer and protein solutions were measured before the membrane immersion to check them and avoid possible fluctuations in the UV apparatus response.

The measurements were performed with the help of a UV–visible spectrometer Lambda 365, PERKIN ELMER, Waltham, MA, USA.

### 2.6. Permeability Measurement

First, membrane compaction was performed, which is the compression of the membrane achieved by applying pressure, so to obtain steady transport characteristics.

In order to perform the compaction, dead-end filtration of ultrapure water was conducted through the membrane placed in an Amicon cell (Series 8010, Merck Millipore, Burlington, MA, USA). For that, the pressure was increased between 0.2 and 1 bar by steps of 0.2 bar, and for each pressure the weight of the permeate was measured every 5 min during 20 min to observe instantaneous flux changes and verify the flux’s stability. At 1 bar, the flux was monitored until it reached almost a steady state.

After that, the pressure was decreased in the same range, and the permeability was determined from the slope of the curve obtained by plotting flux vs. pressure, according to Darcy Law:

(2) $$J_{w}=\frac{L_{p}}{\mu }\;\Delta P$$

where *J_w_* is the water flux (m^3^·m^−2^ ·s^−1^), *L_p_* is the permeability (m), *μ* is the viscosity of the fluid (Pa·s), and Δ*P* is the transmembrane pressure (Pa).

It is important to mention that an area of the membrane larger than that of 0.5 cm^2^ previously employed (3.8 cm^2^) was used during the filtration experiments.

After the evaluation of the pristine membrane permeability, its coating was performed. For this purpose, the cell was emptied of water without removing or touching the compacted membrane, and the copolymer solution was then added. The membrane was left in contact with 4 mL of 5 mg·mL^−1^ copolymer solution during 2 h. Subsequently, the copolymer solution was removed from the cell, and the permeability of the coated membrane was determined in the same way as for the pristine membrane. Indeed, filtration of ultrapure water through the coated membrane was performed by varying the pressure between 0.2 bar and 1 bar. At each pressure, the weight of the permeate was collected every 5 min during 20 min, which was sufficient to reach almost a steady flux. The water flux *J_w_* was then calculated by relating the weighted mass of the permeate to the time and the membrane area (3.8 cm^2^). All experiments were performed at room temperature (22 ± 3 °C).

### 2.7. Dead-End Filtration of HSA

The dead-end filtration of HSA was performed on membranes coated with the different copolymers mentioned in the previous sections. This experiment was carried out by filling the Amicon cell with 10 mL of protein solution at a concentration of 1 mg·mL^−1^ that was filtered through the membrane. The pressure was set at 1 bar, and the weight of the permeate was recorded every minute until almost 3 mL of solution was left in the retentate. The volume reduction factor (VRF), which can be calculated using the formula below, was equal to 3.3:

(3) $$VRF=\frac{V_{0}}{V_{R}}=\frac{V_{0}}{V_{0}-V_{P}}$$

where *V*_0_ is the initial volume of the HSA solution in the cell, while *V_P_* and *V_R_* are the volume of permeate and retentate, respectively. The dead volume that might remain between the membrane and the support and in the permeate pipe was collected with the permeate.

The permeate and the retentate were then collected, and the absorbance of the samples was measured at 280 nm using UV–vis spectroscopy, as explained previously. The absorbance value was recorded, and the protein concentrations in the permeate and retentate were then calculated using the calibration curve (Supplementary Materials Figure S3) indicating the absorbance of HSA at known concentrations.

Further, mass balance was also used during the filtration of the protein solution to calculate the amount of protein adsorbed on the membrane surface, according to the following equation:

(4) $$V_{i}\;C_{i}=V_{P}\times C_{P}+V_{R}\times C_{R}+m_{ads}$$

where *C_P_* and *C_R_* are the HSA concentration in permeate and retentate, respectively, at the end of the dead-end filtration (i.e., for a VRF = 3.3).

All experiments, from membrane compaction to membrane coating and dead-end filtration of HSA, were performed at a room temperature (22 ± 3 °C). The experiments were repeated at least twice to confirm the obtained results.

## 3. Results and Discussions

### 3.1. Static Adsorption of HSA on a PS-b-PAA-Coated PVDF Membrane

Before HSA adsorption was investigated, the PVDF membrane coating with the PS-b-PAA copolymer was analyzed. The copolymer hydrophobic/hydrophilic block ratio and chain length have a substantial role in the copolymer adsorption onto a membrane surface. Thereby, the coating was conducted using copolymers with different sizes (PS_30_-b-PAA_5_, PS_26_-b-PAA_76_ and PS_100_-b-PAA_107_) at the same concentration, which was 5 mg·mL^−1^,. The samples were then prepared for FTIR mapping as explained previously, and maps were generated for the peak at 700 cm^−1^ to assess the copolymer distribution over the membrane surface.

**Figure 1:**
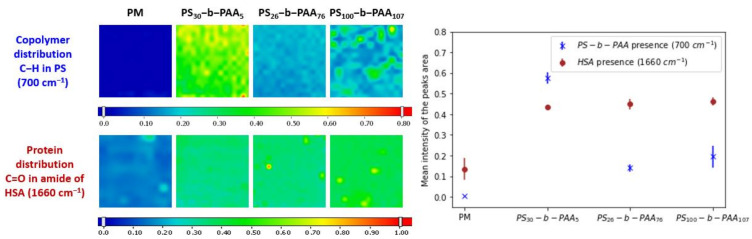
FTIR maps of the coating of the PVDF membranes with the various PS-b-PAA copolymers and of the adsorption of HSA on these membranes; [HSA] = 1 mg·mL^-1^; pH ≈ 7. Also shown are the mean intensities evaluated from the FTIR maps (size of each FTIR panel 5 mm × 5 mm).

The results shown in Figure 1 revealed the attachment of the three copolymers to the membrane surface. This attachment was certainly due to the hydrophobic interactions between the PS block (basically used as the anchoring group) and the PVDF membrane. The maps clearly displayed a higher coverage of the membrane surface when using PS_30_-b-PAA_5_ rather than the other two copolymers. Indeed, as determined from the FTIR analysis, the peak area (700 cm^−1^) exhibited a lower adsorption intensity for the PS_26_-b-PAA_76_-coated membrane than for the PS_30_-b-PAA_5_ coated membrane. Thus, for copolymers with almost the same hydrophobic chain length, the copolymer with the shortest hydrophilic block adhered better to the membrane surface. This means that the hydrophobic interactions between the PVDF membrane and the PS block were diminished by the stronger hydrophilic interactions between the solvent and the PAA segment due to the increase in PAA size. The same trend was reported in previous studies on PS-PEGMA coating on hydrophobic PVDF membranes, which showed that increasing the chain length of the hydrophilic PEGMA block reduced the copolymer adhesion [43,44].

Furthermore, the decrease in adhesion propensity could be also due to the fact that larger PAA moieties introduced increased steric hindrance, blocking the access of PS moieties to the PVDF surface [39,44]. The coverage was greater with PS_100_-b-PAA_107_ than with PS_26_-b-PAA_76_ but still lower than that observed with PS_30_-b-PAA_5_, which might be due to the steric hindrance caused by a large PAA polymer. This suggested that from a certain length of the hydrophilic segment, steric hindrance dominated over the hydrophobic interactions of the PS block with the PVDF membrane.

In the FTIR maps (Figure 1), it appears that the distribution of the copolymer over the membrane surface was not homogeneous. This could be due to heterogeneity of the membrane morphology and to the distribution of the membrane pores. This heterogeneity is represented by the standard deviations of the average values of the intensity, as shown in Figure 1.

Subsequently, the effect of the copolymer size on the adsorption of HSA was also examined using FTIR mapping. The adsorption of HSA on the coated membranes was conducted using a solution with a protein concentration of 1 mg·mL^−1^ at a pH around 7 (pH of PBS). FTIR maps were generated for the peak at 1660 cm^−1^ to determine the presence of HSA over the modified membrane. The maps showing the presence of PS-b-PAA copolymer and HSA protein were generated from the same membrane sample. The FTIR maps (Figure 1) showed that the HSA amount was higher on the coated membranes compared to the pristine one, but it seemed that this increase was not correlated with the copolymer size, since almost the same intensity was obtained for the different copolymers, while high differences were found in the presence of the coating with respect to the PM.

These results indicated the propensity of HSA to adsorb on the PVDF pristine membrane (PM), which could be due to hydrophobic interactions between the membrane and the hydrophobic parts of HSA [35]. In addition, the results demonstrated that the distribution of the protein was not homogeneous on the pristine membrane, which was shown, as stated before, by the standard deviations of the average values of the intensity. This was probably due to heterogeneity in the morphology of the membrane, implying variations in the hydrophobic interactions’ strength over the membrane surface.

In addition, the results displayed a greater propensity of the PS-b-PAA-coated membrane compared to the PM to adsorb HSA. In our previous study on HSA–PAA interactions in solution using small-angle X-ray scattering (SAXS) [32], complexation of HSA with PAA was proved to happen at pH 5 only, while the experiments here were carried out at pH 7. However, other studies reported that the adsorption of BSA on a planar PAA brush layer could take place at pH values higher than 5, even if it reached a maximum at pH around 5, which was attributed to protein charge regulation [24]. Moreover, HSA–PAA binding in solution [27] and HSA adsorption onto nanoparticles grafted with PAA [45] were reported at pH around 7. This adsorption was explained by the release of counter-ions and the attraction between the negatively charged PAA chains and the positive patches carried on the HSA surface [18].

It is important to remember that at pH around 7, the protein and the PAA chains are both negatively charged since HSA has an isoelectric point around 4.7, and PAA has a pKa of 4.5.

Thereby the greater propensity of the PS-b-PAA-coated membrane compared to the PM to adsorb HSA could be due to hydrophobic interactions between the hydrophobic parts of the protein and the accessible PS segments horizontally attached to the surface and not completely hidden by the PAA layer. Indeed, while our work showed a moderate adsorption of HSA on the PVDF pristine membrane, it was reported in the literature that HSA adsorbs strongly onto polystyrene plates and onto polystyrene latex [46].

Another factor could be involved, in addition to the hydrophobic interactions described above, i.e., electrostatic attractions caused by protein charge inversion due to the negative electrostatic potential in the brush [19] or that could take place between positive patches in HSA and the negatively charged PAA, according to a study reported in the literature [18]. In this previous study, simulations showed that PAA could bind to some preferential positive patches on HSA because of specific electrostatic interactions.

### 3.2. Influence of pH on HSA Static Adsorption onto PS-b-PAA-Coated Membrane

In the previous section, the adsorption of HSA on PVDF membranes coated with PS-b-PAA was observed at pH ≈ 7. Meanwhile, it was proven explicitly in our previous work that at this same pH, HSA did not show any affinity for PAA chains in solution [32]. In fact, the binding between the two compounds was found to happen only at pH 5, leading to the formation of HSA–PAA complexes. Wherefore, the adsorption of 1 mg·mL^−1^ of HSA was examined at different pH values (5, 7, and 8) on membranes coated with two copolymers, PS_30_-b-PAA_5_ and PS_100_-b-PAA_107_. As before, FTIR maps were generated first for the peak area at 700 cm^−1^, to confirm that the copolymer was correctly coated onto the membrane surface, and then for the peak area at 1660 cm^−1^ to assess the presence of HSA. The results (Supplementary Materials Figure S4) confirmed that both copolymers were correctly coated and showed their presence all over the membrane surface. Indeed, the evaluated mean intensities of the peak area at 700 cm^−1^ were almost the same for the different coated samples. That allowed us to compare the adsorption of HSA at different pH values on these same samples.

**Figure 2:**
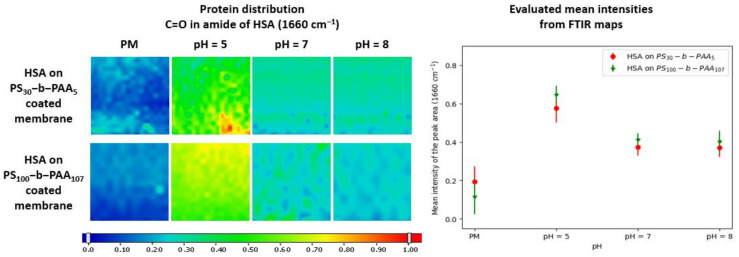
FTIR maps of the adsorption of HSA at different pH values (5, 7, and 8) on PVDF membranes coated with PS_30_-b-PAA_5_ and PS_100_-b-PAA_107_ copolymers and evaluated mean intensities of the peak area at 1660 cm^−1^.

**Figure 3:**
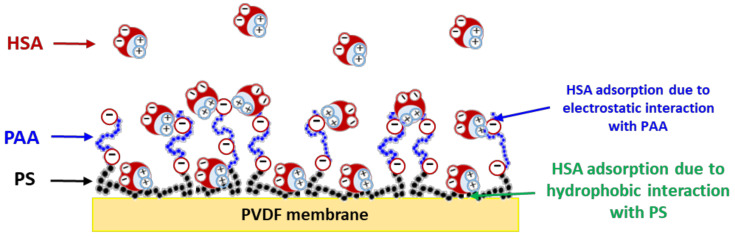
Illustration of HSA adsorption onto a PS-PAA-coated membrane due its hydrophobic interactions with the accessible PS and to electrostatic interactions with PAA brushes.

The maps shown in Figure 2 indicated the presence of HSA over the whole range of the studied pH values, but its adsorption was found to be greater at pH 5, and this was confirmed by the mean intensities of the peak area at 1660 cm^−1^ (Figure 2). An intensity of 0.58 was obtained at pH 5, whereas an intensity of 0.37 was measured at pH 7 and 8 on membranes coated with PS_30_-b-PAA_5_. As for membranes coated with PS_100_-b-PAA_107_, the intensity was 0.64 at pH 5 and 0.41 and 0.40 at pH 7 and 8, respectively. This is consistent with the behavior of BSA reported in a previous study [24], with the maximum of adsorption on a planar PAA brush layer achieved near the point of zero charge (pzc) of the protein. Thereby, at pH 5, the protein and the PAA chains are both slightly negatively charged, as stated previously. However, even though the protein has a slightly negative overall charge at this pH, which is not so far from the protein IP, it can carry numerous positives patches on its surface [18], as shown in Figure 3.

As stated previously, the behavior at pH 5 was probably due to the same hydrophobic interactions as those suspected at pH 7 (previous section), with the addition of electrostatic attractions, which we revealed in our study in solution at pH 5 [32], as depicted in Figure 3. Thus, HSA adsorption driven by the attractive electrostatic interactions with PAA could increase the total amount of HSA adsorbed onto the membrane surface.

**Figure 4:**
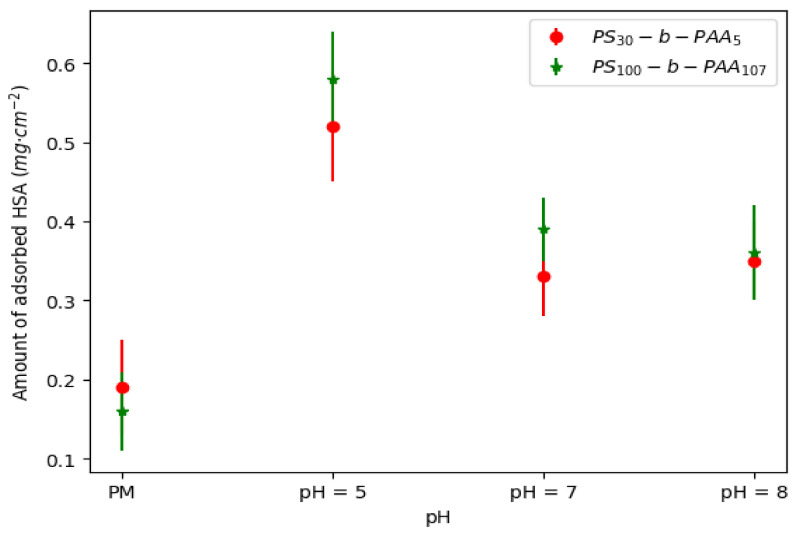
The adsorbed amount of HSA on PVDF membranes coated with PS_30_-b-PAA_5_ and PS_100_-b-PAA_107_ copolymers at different pH values; [HSA] = 1 mg·mL^−1^.

Subsequently, the adsorbed amount of HSA was estimated by mass balance before and after immersion of the coated membranes in the protein solution (concentrations determined by UV–visible spectroscopy). Since adsorption took place during the immersion of the membrane, the adsorbed amount of HSA was calculated by considering the area of both sides of the membrane (1 cm^2^). Even though the adsorbed amounts of HSA were estimated on both sides of the membrane, the results plotted in Figure 4 and summarized in Supplementary Materials Table S2 confirmed a maximum of HSA adsorption at pH 5.

This outcome corroborated the hypothesis suggesting that HSA adsorption at pH 5 was controlled by two forces, i.e., the hydrophobic interactions of HSA with PS and an increased electrostatic attraction to PAA. The HSA–PAA interaction evidenced in solution at pH 5 was here responsible for an increase in the adsorbed HSA amount of ca. 53%.

### 3.3. Ionic Strength Effect on HSA Static Adsorption onto PS-b-PAA-Coated Membrane

Since we suggested that the increase in HSA adsorption at pH 5 could be due to increased attractive electrostatic interactions, we varied the salt concentration by adding the desired amount of NaCl to the protein solutions. The ionic strength was increased from 0 M of added salts (0.05 M provided by the buffer) to 0.5 M final concentration of added salts, and the pH was set at 5 using a mixture of sodium citrate di-hydrate and citric acid.

**Figure 5:**
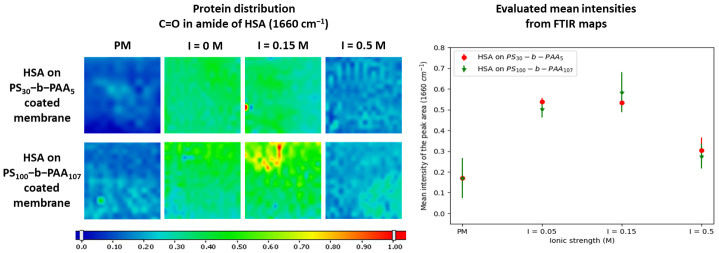
FTIR maps of the adsorption of HSA at different ionic strengths (0.05, 0.15, and 0.5 M) on PVDF membranes coated with the PS_30_-b-PAA_5_ and PS_100_-b-PAA_107_ copolymers, as well as evaluated mean intensities of the peak area at 1660 cm^−1^.

Coating was performed using PS_30_-b-PAA_5_ and PS_100_-b-PAA_107_ at a copolymer concentration of 5 mg·mL^−1^. After that, the modified membranes were immersed in 1 mg·mL^−1^ of HSA solution, before being dried and characterized using FTIR mapping. The maps were then treated the same way as before, and the mean intensities of the peaks’ areas at 700 cm^−1^ and 1660 cm^−1^ were evaluated. The FTIR maps and evaluated mean intensities shown in Supplementary Materials Figure S5 confirmed that both copolymers were coated all over the membrane surface. We found a decrease in HSA adsorption with the increase in ionic strength (Figure 5). For membranes coated with PS_30_-b-PAA_5_, the intensity was 0.53 at ionic strengths (I) of 0.05 M and 0.15 M, whereas it was 0.3 at I = 0.5 M. As for the membranes modified with PS_100_-b-PAA_107_, the intensity was 0.51 and 0.58 at the salt concentrations of 0.05 M and 0.15 M, respectively, whereas it was 0.28 at I = 0.5 M.

This outcome is consistent with what was shown regarding the interaction between HSA and PAA in solution, which was found to take place at ionic strength ≤0.15 M [32]. This confirmed in some way the attractive electrostatic nature of the interactions between the few positively charged protein patches and the negatively charged PAA layer. This is also consistent with what was stated previously regarding the interactions between HSA and PAA [18], on the basis of simulations that revealed that PAA can bind to some preferential positive patches on HSA because of specific electrostatic interactions. In fact, attractive interactions will be screened by the addition of salts, and the range of the attractive interactions will be reduced.

Therefore, electrostatic repulsion due to the negative overall charge of both compounds overcame the localized attractive forces and reduced the adsorption (as shown at I = 0.5 M in Figure 5). The intensity was around 0.3 at I = 0.5 M, even at pH 5, which was lower than the intensity of around 0.4 at pH 7 and I = 0.15 M. This confirmed that the adsorption of HSA at pH 7 on a PVDF membrane coated with PS-b-PAA was not due only to hydrophobic interactions. It proved that electrostatic interactions played a role even at pH 7, and this role became more important at pH 5 due to stronger electrostatic attractive forces. Overall, this confirmed the ionic strength dependence of protein–polyelectrolyte interactions revealed in many previous studies [19,24,47].

### 3.4. Dead-end Filtration of HSA through PS-b-PAA-Coated Membranes at Neutral pH

The permeability of the coated membranes was calculated as explained in Section 2.6 and was compared to that of the pristine membrane to evaluate the variation of the transport properties due to the coating. The experiments were conducted using PS_30_-b-PAA_5_, PS_26_-b-PAA_76_, and PS_100_-b-PAA_107_ in order to explore the copolymer size influence on the permeability of the membrane. The plots of flux versus pressure, from which the permeability was calculated after coating with each copolymer, are shown in Supplementary Materials Figure S6, and the results are summarized in Table 2. The permeability decreased with the increase in size of the copolymer coated over the membrane. The permeability was reduced by around 31% after coating with PS_30_-b-PAA_5_ and by 48% after coating with PS_26_-b-PAA_76_, whereas the decline was of 65% after PS_100_-b-PAA_107_ coating. It seemed that longer chains blocked the passage of water through the membrane by creating additional resistance in the system.

**Figure 6:**
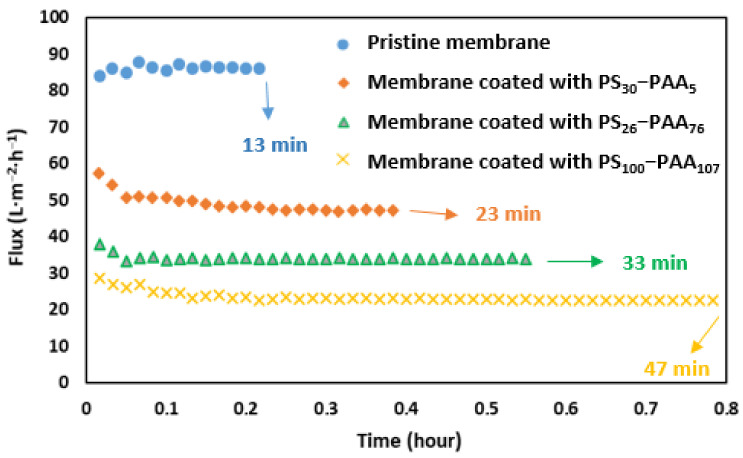
Flux evolution versus time during HSA filtration at 1 bar through pristine and modified membranes coated with different PS-b-PAA copolymers, [HSA] = 1 mg·mL^−1^; pH = 7.

Dead-end filtration of HSA was carried out once the coating of the compacted membranes had been successfully performed and the permeability had been measured. For that, an Amicon cell was filled with 10 mL of HSA solution, and filtration was performed at 1 bar, until around 3 mL of the solution was left in the retentate (VRF = 3.3). As stated in the Section 2, during filtration, the permeate mass was recorded every minute, so that the permeate flux could then be calculated. The flux evolution during HSA filtration through every modified membrane was plotted versus time and compared to that obtained for the PM, as shown in Figure 6.

For the same volume of permeate (≈7 mL), the filtration lasted 13 min for the PM, while it lasted 23 min, 33 min, and 47 min for the membranes coated with PS_30_-b-PAA_5_, PS_26_-b-PAA_76_, and PS_100_-b-PAA_107_, respectively. This was expected, since we showed above that the permeability of the membrane decreased with the size of the copolymer.

In Figure 6, one can observe that the flux decreased slightly in the beginning before becoming almost stable, irrespective of the copolymer. This could be due to the adsorption of HSA on the membrane, which reduced the flux. However, the pristine membrane did not exhibit the same behavior, and the flux rate was almost stable between 84 and 87 L·m^2^·h^−1^. Thereby, one can notice that the adsorption of HSA on the pristine membrane did not affect the membrane’s transport properties, in contrast with what observed for the modified ones. The flux was slightly reduced by HSA adsorption on the PS-b-PAA-coated membranes. It decreased from 57 to 47 L·m^2^·h^−1^ for the membrane coated with PS_30_-b-PAA_5_, from 38 to 33 L·m^2^·h^−1^ for the PS_26_-b-PAA_76_-coated membrane, and from 28 to 23 L·m^2^·h^−1^ for the PS_100_-b-PAA_107_-coated membrane. This corresponded to reduction ratios of 0.82, 0.87, and 0.79, respectively, which means that the flux reduction was almost the same for the three copolymers and did not seem to be related to the copolymer size. Once this adsorption was completed, there was no significant variation in the flux, suggesting the absence of other clogging mechanisms over long periods.

**Figure 7:**
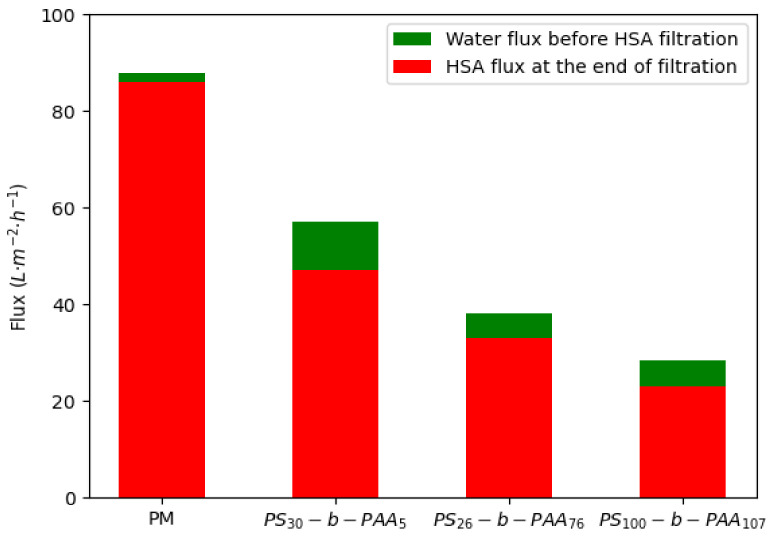
The water flux *J_w_* after surface modification by different PS-b-PAA copolymers and the flux of the HSA solution *J* at the end of filtration through the modified membranes.

These results mainly indicated that the reduction in permeability due to the protein adsorption was less substantial than that due to the deposition of the copolymer. This could be illustrated by plotting the flux rates after surface modification by different PS-b-PAA copolymers and at the end of HSA filtration through the modified membranes (Figure 7).

**Figure 8:**
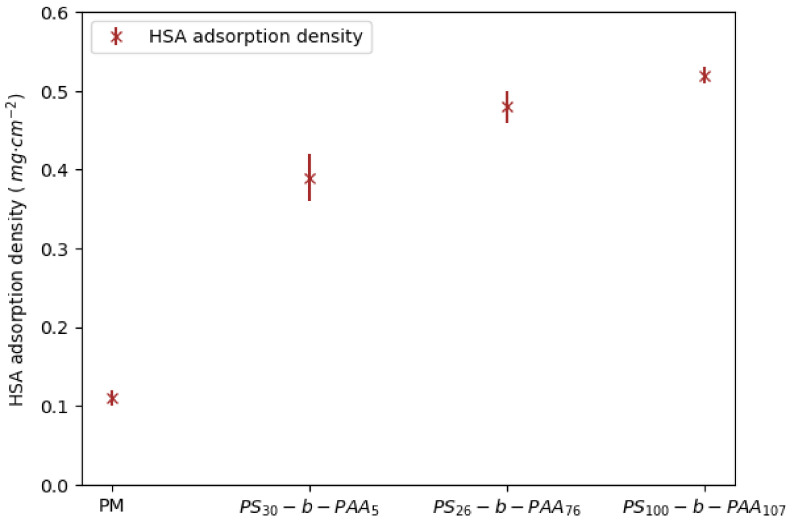
HSA adsorption density on PM as well as on PS-b-PAA-coated membranes after dead-end filtration; pH = 7.

The concentration of HSA in the permeate and in the retentate collected at the end of filtration was estimated using UV–visible spectroscopy. The amount of protein adsorbed on the membrane surface was calculated by mass balance using Equation (4). The results are summarized in Supplementary Materials Table S3 and in Figure 8.

The adsorption density of HSA onto the membrane surface was around 0.11 mg·cm^−2^, which is comparable to the protein adsorption density of 0.15 mg·cm^−2^ for a commercial membrane reported by the supplier, as shown in Supplementary Materials Table S3. The HSA adsorption density increased to 0.39 mg·cm^−2^ on the membrane coated with PS_30_-b-PAA_5_ and reached 0.48 mg·cm^−2^ and 0.52 mg·cm^−2^ on those coated with PS_26_-b-PAA_76_ and PS_100_-b-PAA_107_, respectively.

Hereafter, the presence of the copolymer over the membrane surface was checked, and the adsorption of HSA onto the membranes was confirmed using FTIR, as shown in Supplementary Materials Figure S7.

As discussed in the previous sections on static adsorption, the presence of the copolymer and the size of the PS blocks increased the amount of HSA accumulated on the membrane surface. It was reported previously that HSA adsorption at pH around 7 (which was the HSA solution’s pH during filtration) was driven by a combination of hydrophobic interactions and electrostatic attractions (Figure 3). It can also be noted that the adsorption density resulting from filtration was higher than that observed after static adsorption (Figure 4), with an increase in adsorption density between +30% and +45%. In addition, one can imagine that the increase in HSA adsorption during dead-end filtration was due to an adsorption enhancement by the permeate flux.

### 3.5. Dead-end Filtration of HSA through PS-b-PAA-Coated Membranes at pH 5

In Section 3.2, we showed that HSA static adsorption on PS-b-PAA-coated membranes was greater at pH 5. In this section, dead-end filtration of HSA was repeated at pH 5 through a PVDF membrane coated with PS_100_-b-PAA_107_ in order to examine the effect of pH on HSA adsorption in a dynamic mode. The membrane transport properties were also investigated before and after dead-end filtration, and the results are reported in Supplementary Materials. The procedure of compaction, coating, and filtration was used as explained previously, changing the pH of the HSA solution during filtration. The presence of the copolymer and the protein was, as usual, assessed using FTIR mapping (Supplementary Materials Figure S8). Moreover, the concentration of HSA in the permeate and in the retentate was estimated by UV–visible spectroscopy, and the amount of HSA adsorbed onto the membrane surface was determined by mass balance. In order to explore the effect of pH, the results obtained at pH 5 were compared to those obtained earlier at pH 7 for the same copolymer (Table 3).

**Figure 9:**
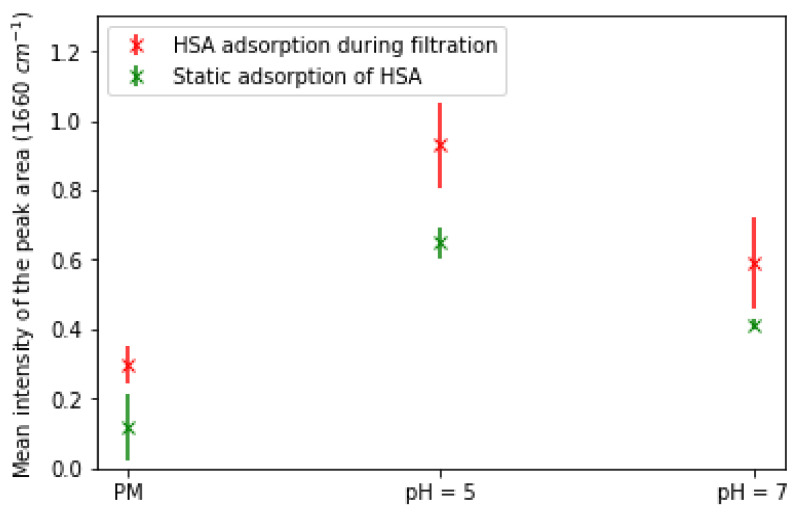
Evaluated mean intensities of the peak area at 1660 cm^−1^ showing the adsorption of HSA onto PS_100_-b-PAA_107_-coated membranes at pH 5 and 7 during filtration compared to those obtained for the static adsorption of HSA in the same conditions; [HSA] = 1 mg·mL^−1^.

These results confirmed the observation that HSA deposition onto the membrane surface at pH 5 was slightly larger. Indeed, the adsorption density of HSA was found to be 0.59 mg·cm^−2^ at pH 5 and 0.52 mg·cm^−2^ at pH 7. Yet, this increase (12%) is not sufficient to definitively establish an increase in protein adsorption at pH 5, especially as the method used (UV–visible) previously showed standard deviations of around 10% or more. Thus, to confirm the increase in HSA adsorption, we resorted to calculating the mean intensities from the FTIR maps (Supplementary Materials Figure S8), as explained previously. The obtained results shown in Figure 9 were then compared to the mean intensities obtained for the static adsorption of HSA onto a PS_100_-b-PAA_107_-coated membrane at pH 5 and pH 7.

These results showed effectively that HSA adsorption during filtration at pH 5 was greater than at pH 7; the mean intensity was around 0.93 and 0.59 at pH 5 and 7, respectively. It can also be noticed in Figure 9 that HSA adsorption during filtration was clearly greater than that measured during static adsorption. Indeed, the evaluated intensities during static adsorption of HSA were 0.64 and 0.41 at pH 5 and 7. This outcome sustains the conclusion that HSA adsorption during filtration was enhanced by the flow through the membrane.

## 4. Conclusions

The interaction of proteins with polymer surfaces has major implications in the biomedical field, as stated in the introduction. In the case of biomaterials coming into direct contact with blood, implementing surface coatings may help to control the interaction with the blood components. For that, it is substantial to identify and describe the determinants of the interaction of coatings with the major proteins of blood plasma such as HSA, interaction that would condition their use. Thus, this study reports several findings that allow us to better understand the interactions between a PS-b-PAA-coated PVDF membrane and HSA, discussing the factors that determine them.

First, coating via physisorption was shown in this study to be an effective technique for the modification of PVDF membranes. In fact, the hydrophobic interactions between the PS blocks and the PVDF membranes were clearly strong enough to keep the copolymer layer coated on the membrane surface. This then allowed the investigation of the adsorption of the HSA protein onto the coated membranes using mainly FTIR mapping, which was proved to be a powerful tool to assess the presence of both coating and HSA on the surface at the same time. Afterward, the adsorption of HSA on PS-b-PAA-coated membranes was revealed to be correlated with the pH and the ionic strength. Indeed, both FTIR mapping and mass balance calculations using UV–visible spectroscopy, indicated a great adsorption of HSA at pH close to 5 and at low ionic strength (≤0.15M). This adsorption was then shown to be reduced at higher pH and ionic strength. That revealed the involvement of electrostatic attraction between HSA and PAA in this adsorption, in addition to the hydrophobic interactions that could happen between HSA and the PS blocks. Finally, dead-end filtration sustained these results, since the deposition of HSA was found to increase again at pH 5, even though the amount of HSA on the membrane surface was this time noticed to be greater than that measured during the static adsorption of the protein.

This study confirmed the role of the specific interactions between HSA and PAA in HSA absorption depending on physicochemical conditions (pH and ionic strength), proved previously in solution [32], and the possibility of exploiting this specificity in membrane separation processes.

## Figures and Tables

**Table 1:** Main characteristics of the copolymers used: molar mass, polydispersity, hydrodynamic diameter *D_h_*, and the estimated radius of gyration *R_g_*.

Formula	M_n_ (kDa) 1Da = 1 g·mol^−1^	PDI M_w_/M_n_	*D_h_* * (nm)	R_g_ * (nm)
PS_30_-b-PAA_5_	PS ≈ 27–31 PAA ≈ 4–6 M_n average_ ≈ 31–37	≤1.3	5.1	3.8
PS_26_-b-PAA_76_	PS ≈ 26 PAA ≈ 76 M_n average_ ≈ 102	≤1.2	8.3	6.2
PS_100_-b-PAA_107_	PS ≈ 100 PAA ≈ 107 M_n average_ ≈ 207	≤1.1	12.9	9.7

* *D_h_* was obtained by DLS measurements and was then used to estimate *R_g_* (Supplementary Materials).

**Table 2:** Permeability of membranes coated with different PS-b-PAA copolymers compared to that of the pristine membrane, as well as decline percentage caused by the coating.

Sample	Permeability (L·m^−2^·h^−1^·bar^−1^)	Permeability Decline (%)
Pristine membrane	86	__
Membrane coated with PS_30_-b-PAA_5_	59	31
Membrane coated with PS_26_-b-PAA_76_	44	48
Membrane coated with PS_100_-b-PAA_107_	30	65

**Table 3:** Comparison of HSA adsorption on PVDF membranes coated with PS_100_-b-PAA_107_ during dead-end filtration at different pH values (5 and 7).

Sample	Concentration of HSA in Permeate (mg·mL^−1^)	Concentration of HSA in Retentate (mg·mL^−1^)	Mass of HSA Adsorbed (mg)	Adsorption Density (mg·cm^−2^)
pH = 7	0.49	1.53	1.98	0.52
pH = 5	0.51	1.39	2.26	0.59

## Data Availability

Data are contained within the article and Supplementary Materials.

## Supplementary Materials

The following supporting information can be downloaded at: https://www.mdpi.com/article/10.3390/membranes13120886/s1, Figure S1: ATR spectra of PVDF pristine membrane, pure PS-b-PAA copolymers, and native HSA protein, Figure S2: FTIR spectra obtained after mapping on PM and coated membrane with the adsorption of HSA, illustrating the calculation of the area of chosen peaks. Figure S3: Calibration curve of the absorbance obtained at 280 nm by UV-visible spectroscopy, as a function of HSA concentration, Figure S4: FTIR maps for the coating of PVDF membranes with PS30-b-PAA5 and with PS100-b-PAA107 copolymers at different pH values (5, 7, and 8), as well as the evaluated mean intensities of the peak area at 700 cm^−1^, Figure S5: FTIR maps for the coating of PVDF membranes with PS30-b-PAA5 and with PS100-b-PAA107 copolymers at different ionic strengths (0.05, 0.15, and 0.5 M), as well as the evaluated mean in-tensities of the peak area of 700 cm^−1^, Figure S6: Plots of the water flux vs. pressure after coating with each copolymer from which the permeability was calculated, Figure S7: FTIR maps generated at 700 cm^−1^ and 1660 cm^−1^, showing the presence of the coating layer and that of the protein after HSA dead-end filtration through the PVDF membrane coated with different PS-b-PAA (pH ≈ 7; [HSA]= 1 mg·mL−1; I ≈ 0.15 M), Figure S8: FTIR maps, generated at 700 cm^−1^ and 1660 cm^−1^, showing the presence of the coating layer and that of the protein after HSA dead-end filtration through a membrane coated with PS100-b-PAA107 (pH = 5 and 7; [HSA] = 1 mg·mL^−1^), Figure S9: Plot of the water flux versus pressure from which the permeability was calculated for membranes coated with PS100-b-PAA107, and evolution of HSA solution flux versus time during HSA filtra-tion (pH = 5) through this same membrane, Table S1: Absorption bands of functional groups in PS-b-PAA copolymers and those in HSA protein, Table S2: The amounts of HSA adsorbed onto PVDF membranes coated with PS30-b-PAA5 and PS100-b-PAA107, estimated using UV-visible spectroscopy, Table S3: Calculations of the amount of HSA adsorbed on the membrane surface and/or structure by mass balance.
