# Supplementary material for: pH-Dependent Adsorption of Human Serum Albumin Protein on a Polystyrene-Block–Poly(acrylic acid)-Coated PVDF Membrane
Source: Membranes (Basel). 2023 Nov 22;13(12):886. doi: 10.3390/membranes13120886 (PMC10744623; doi:10.3390/membranes13120886)
Supplement: Supplementary file 1 [file membranes-13-00886-s001.zip › membranes-2705443-supplementary.pdf]

Supplementary material for

pH-Dependent Adsorption of Human Serum Albumin Protein on a Polystyrene-Block–Poly(acrylic acid)-Coated PVDF Membrane

Charaf Eddine Merzougui *, Pierre Aimar, Patrice Bacchin and Christel Causserand *

S1. Copolymers characterization

The sizes of the copolymers were measured using dynamic light scattering (DLS) to evaluate the hydrodynamic diameter (D_h). Afterward, the D_h was used to estimate the copolymer's radius of gyration using the equation below:

$$R_h = 0{,}665 \times R_g$$

where $R_h = D_h/2$ is the hydrodynamic radius and R_g is the radius of gyration. The obtained values of D_h and R_g are reported in Table 1 of the main article.

S1.1. ATR-FTIR for membrane surface characterization

FTIR mapping is the main technique used to characterize the modified membrane surface; thus, the spectra of the PVDF pristine membrane and the pure copolymers or protein were first identified using ATR. These spectra were then compared in order to differentiate the peaks representing the chemical structure of the copolymer and the protein, as shown in Figure S1.

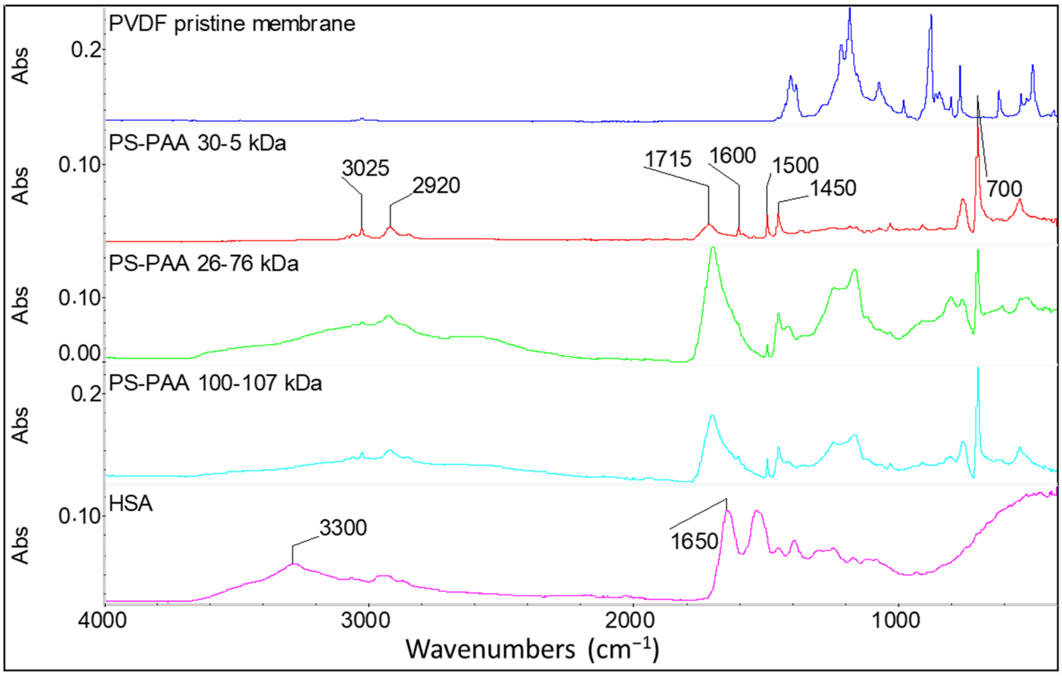

Figure S1. ATR spectra of PVDF pristine membrane, pure PS-b-PAA copolymers, and native HSA protein.

Based on the ATR spectra shown in Figure S1, it can be noticed that the PS group and the copolymer backbone can be easily seen for all PS-b-PAA copolymers. In fact, whether C- H deformation and C=C stretching of the aromatic in PS, =C-H stretching in PS, or -C-H stretching in the copolymer backbone occurred, they are all observable in the ATR spectra, and their peaks are summarized in Table S1**.** As for the hydrophilic group, ideally,

the spectrum of carboxylic acid in PAA should have strong absorption for O-H stretching at 3300-2500 cm^{-1} (broad peak), and for C=O stretching at 1700 cm^{-1}. Thus, these peaks are observable for all of the PS-b-PAA copolymers.

Table S1. Absorption bands of functional groups in PS-b-PAA copolymers and those in HSA protein.

Absorption (cm^{-1})	Functional groups of PS-b-PAA
3300-2500	O-H stretching in PAA
3105-3000	=C-H stretching in PS
3000-2850	-C-H stretching in the polymer backbone
1720-1706	C=O stretching in PAA
1600, 1500, 1450	C=C stretching of the aromatic in PS
700	C-H deformation of monosubstituted aromatic in PS
**Absorption (cm^{-1})**	**Functional groups of proteins**
3500-3070	N-H stretching in the amide
1680-1630	C=O stretching in the amide

As for the protein, the general structure is intricate, but it is known that it is composed of amino acids connected by peptide bonds (amide). Therefore, notable peaks can be observed in their spectra at 3500-3070 cm^{-1}, which is attributed to the amide N-H stretching vibration, and at 1680-1630 cm^{-1} due to the amide C=O stretching, as shown in Table S1.

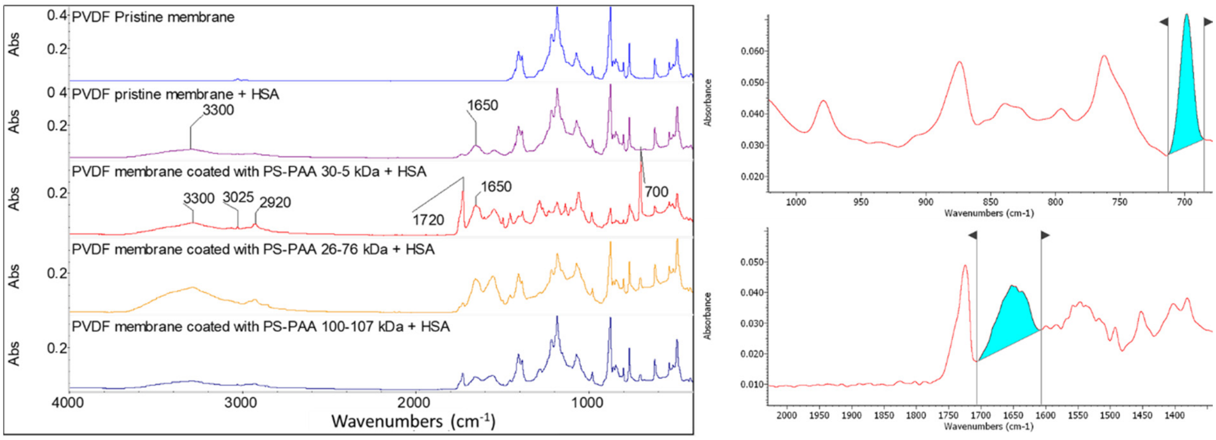

Figure S2. FTIR spectra obtained after mapping on PM and coated membrane with the adsorption of HSA, illustrating the calculation of the area of chosen peaks.

Once the surface modification or the protein adsorption was complete, FTIR mapping was performed. Then, from the obtained FTIR spectra, the peak of interest was chosen and its area was measured (Figure S2) to generate chemical maps, which were color-coded according to absorption peak intensity from blue (lowest intensity) to red (highest intensity). A higher peak intensity indicates more coating or protein presence on the surface.

S2. Calibration curve for UV-visible analysis

In order to evaluate the amount of the protein that was adsorbed onto the membrane using UV-visible spectroscopy, a calibration curve of the absorbance versus the concentration of HSA was first established (Figure S3). For this purpose, the absorbances of different standard solutions with known concentrations of copolymers were measured at 280 nm.

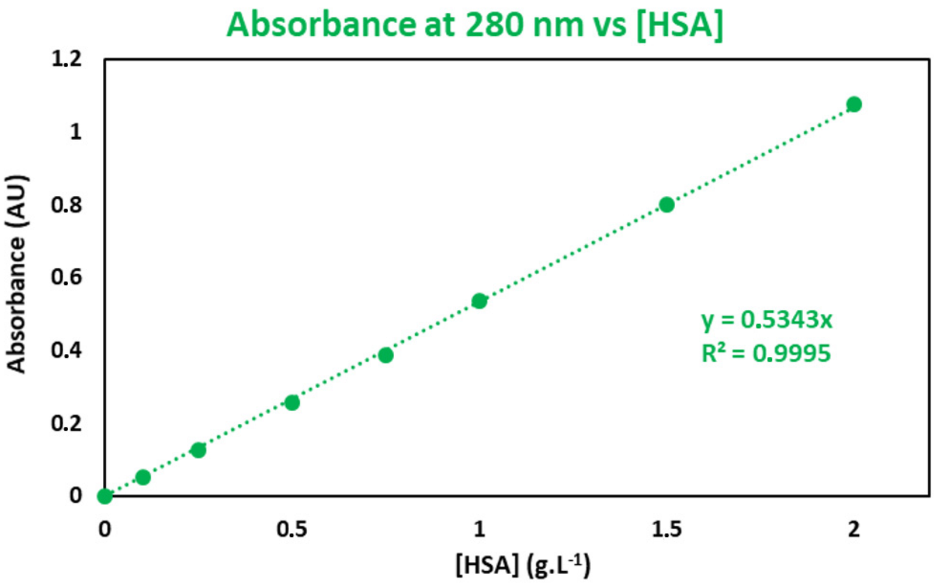

Figure S3. Calibration curve of the absorbance obtained at 280 nm by UV-visible spectroscopy, as a function of HSA concentration.

S3. Influence of pH on HSA adsorption onto membrane coated with PS-b-PAA

The FTIR maps were first generated for the peak area at 700 cm^{-1} to confirm that the copolymer had been correctly coated onto the membrane surface. The obtained results (Figure S4) confirm that both copolymers were correctly coated and show their presence all over the membrane surface. Indeed, for the coating of both copolymers, the evaluated mean intensities of the peak area at 700 cm^{-1} were almost the same for the different coated samples. This allowed us to compare the adsorption of HSA at different pH values on these same samples.

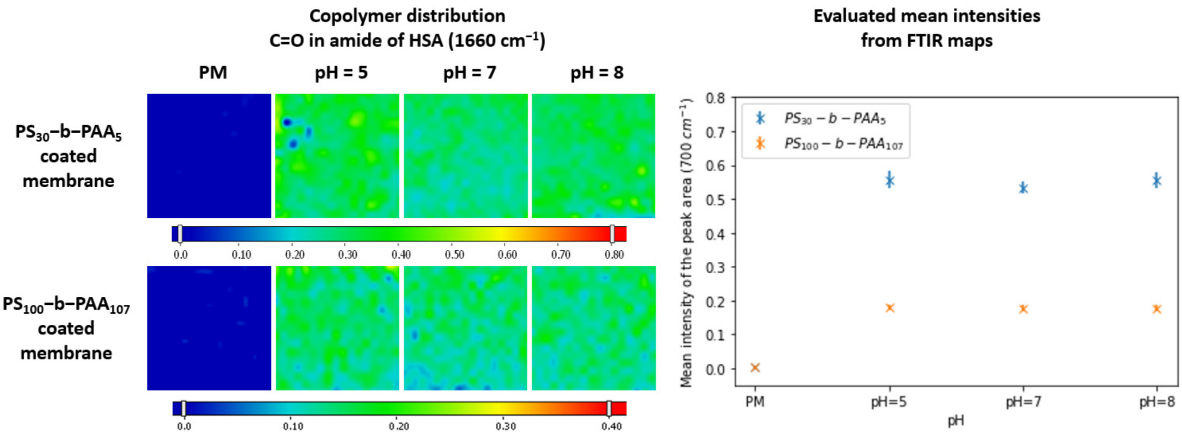

Figure S4. FTIR maps for the coating of PVDF membranes with PS30-b-PAA5 and with PS100-b-PAA107 copolymers at different pH values (5, 7, and 8), as well as the evaluated mean intensities of the peak area at 700 cm^{-1}.

The adsorbed amount of HSA was estimated by difference of mass before and after the immersion of the coated membranes in protein solution using UV-visible spectroscopy. As explained in the main article, the initial concentrations were also evaluated for more reliable measurement. Since the adsorption was carried out by immersion, the adsorbed amount of HSA was calculated by considering the areas of both sides of the membrane (1 cm^2).

Table S2. The amounts of HSA adsorbed onto PVDF membranes coated with PS30-b-PAA5 and PS100-b-PAA107, estimated using UV-visible spectroscopy.

pH	Initial concentration ($mg \cdot mL^{-1}$)	Final concentration ($mg \cdot mL^{-1}$)	Coating density ($mg \cdot cm^{-2}$)	Standard deviation ($mg \cdot cm^{-2}$)
**HSA adsorption on PVDF membranes coated with PS30-b-PAA5**				
PM (pH=7)	1.15	0.96	0.19	0.06 (32%)
5	1.15	0.63	0.52	0.07 (13%)
7	1.15	0.82	0.33	0.05 (15%)
8	1.15	0.8	0.35	0.05 (14%)
**HSA adsorption on PVDF membranes coated with PS100-b-PAA107**				
PM (pH=7)	1.15	0.99	0.16	0.05 (31%)
5	1.15	0.57	0.58	0.06 (10%)
7	1.15	0.76	0.39	0.04 (10%)
8	1.15	0.79	0.36	0.06 (17%)

Even though the adsorbed amounts of HSA were estimated for both sides of the membrane, the results summarized in Table S2 confirm the adsorption of HSA onto the coated membranes in higher amounts at a pH value of 5.

S4. Effect of ionic strength on HSA adsorption onto membranes coated with PS-b-PAA

As previously mentioned, the FTIR maps were generated first for the peak area at 700 cm^{-1} to confirm that the copolymer had been correctly coated onto the membrane's surface. Whether from FTIR maps or from the evaluated mean intensities shown in Figure S5, it can be confirmed that both copolymers were coated all over the membrane surface.

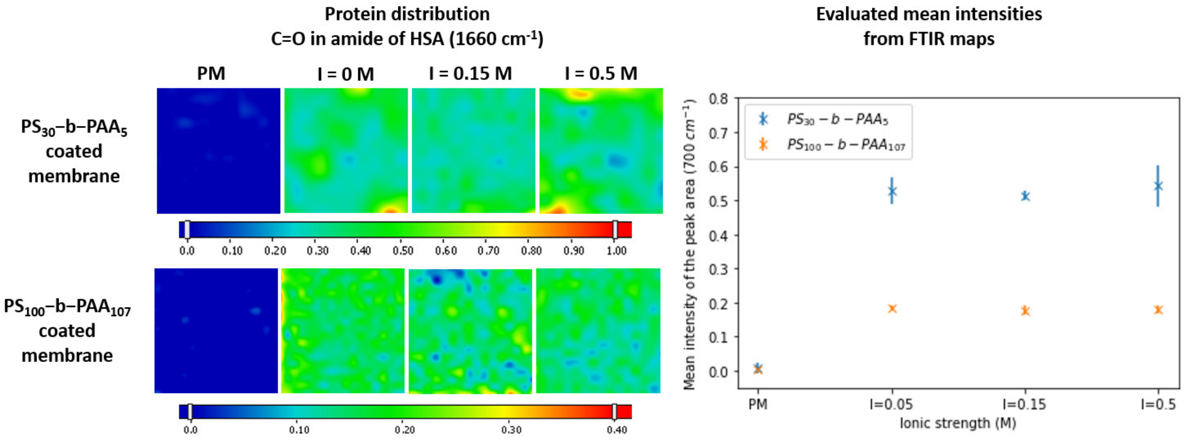

Figure S5. FTIR maps for the coating of PVDF membranes with PS30-b-PAA5 and with PS100-b-PAA107 copolymers at different ionic strengths (0.05, 0.15, and 0.5M), as well as the evaluated mean intensities of the peak area of 700 cm^{-1}.

S5. Permeability of PS-b-PAA coated membrane

The permeability of the coated membranes was calculated as explained in the methods section of the main article, and it was compared to that of the pristine membrane to evaluate the deviation of transport properties due to coating. Plots of the flux versus pressure from which the permeability was calculated after coating with each copolymer are shown in Figure S6.

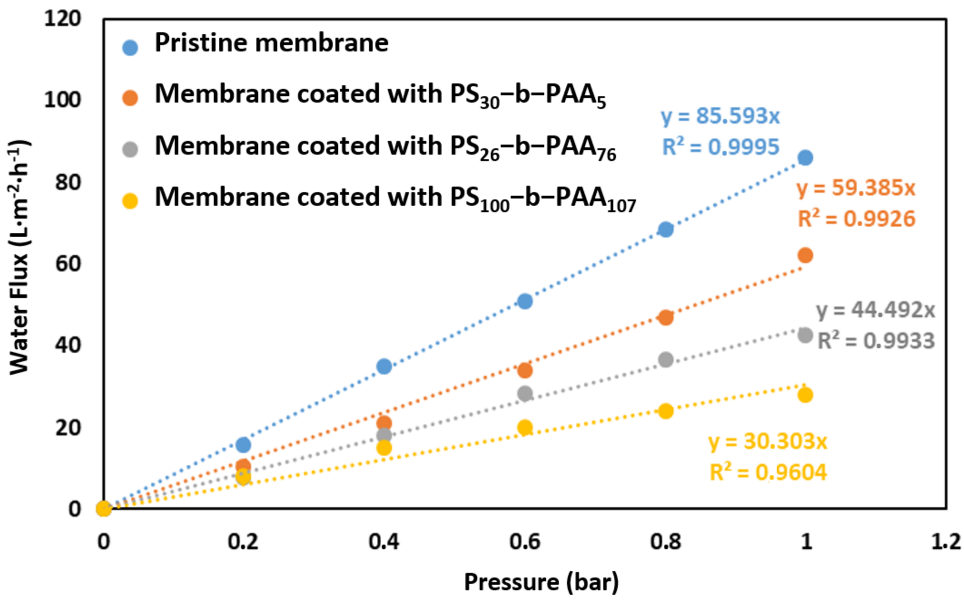

Figure S6. Plots of the water flux vs. pressure after coating with each copolymer from which the permeability was calculated.

S6. HSA adsorption on PS-b-PAA-coated membrane during filtration

The concentration of HSA in the permeate and in the retentate collected at the end of the filtration process was estimated using UV-visible spectroscopy and is summarized in SI-Table S3.

Table S3. Calculations of the amount of HSA adsorbed on the membrane surface and/or structure by mass balance.

Sample	Concentration of HSA in permeate ($mg \cdot mL^{-1}$)	Concentration of HSA in retentate ($mg \cdot mL^{-1}$)	Mass of HSA adsorbed (mg)	Adsorption density ($mg \cdot cm^{-2}$)
**Pristine membrane**	0.92	1.05	0.41	0.11
**Membrane coated with PS30-b-PAA5**	0.74	1.11	1.49	0.39
**Membrane coated with PS26-b-PAA76**	0.58	1.37	1.83	0.48
**Membrane coated with PS100-b-PAA107**	0.49	1.53	1.98	0.52

Thus, the amount of protein adsorbed on the membrane's surface was evaluated by mass balance using Equation 4, which is given in the main article. The outcomes of these calculations are summarized in Table S3.

Once the filtration was complete, the presence of the copolymer as well as the HSA over the membrane surface was then confirmed using FTIR, as shown in Figure S7. The results demonstrate that the three PS-b-PAA copolymers were correctly coated on the

PVDF membrane, and that they were not removed after filtration operation. The results confirm then the adsorption of HSA on the pristine membrane was likely due to hydrophobic interactions between the hydrophobic parts of protein and the PVDF membrane. The deposition of HSA seems, then, to be greater on the coated membrane. Furthermore, a visual analysis of the FTIR maps revealed that it slightly increased with the increase in copolymer size.

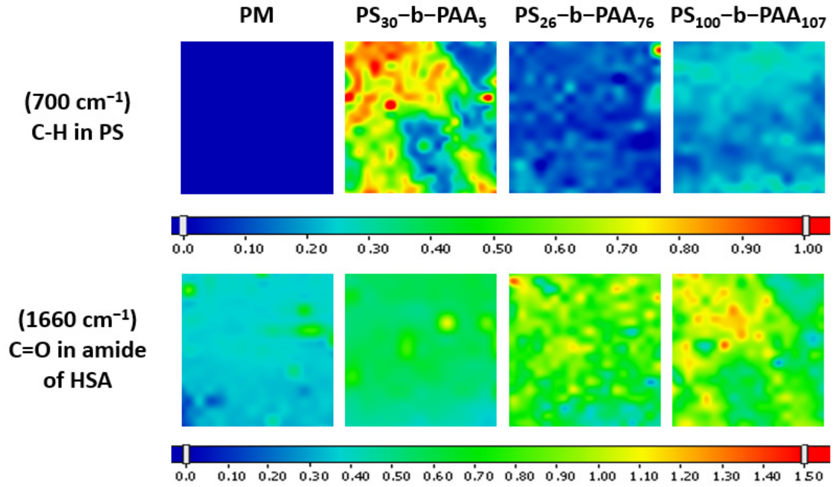

Figure S7. FTIR maps generated at 700 cm^{-1} and 1660 cm^{-1}, showing the presence of the coating layer and that of the protein after HSA dead-end filtration through the PVDF membrane coated with different PS-b-PAA (pH ≈ 7 ; [HSA]= 1 mg·mL^{-1}; I ≈ 0.15M).

S7. Dead-end filtration of HSA at pH 5 through PS-b-PAA coated membrane

The presence of the copolymer and that of the protein were, as usual, assessed using FTIR mapping, and the generated maps were compared to those obtained earlier at pH 7 for the same copolymer in order to explore the effect of pH on HSA adsorption. The FTIR maps shown in Figure S8 first confirm the presence of the copolymer all over the membrane surface, which allows us to discuss the deposition of HSA onto it. In fact, the FTIR maps show a greater adsorption of HSA at pH 5, which is the same trend revealed previously during the static adsorption study. This is probably due to the increase in electrostatic attraction between the negatively charged PAA and the positive patches carried on the HSA surface after the reduction in its negative overall charge when the pH was reduced to 5.

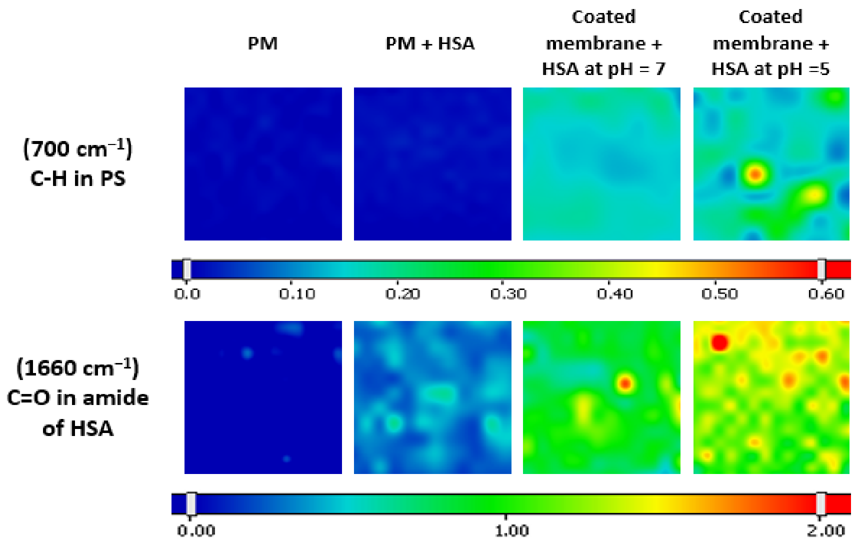

Figure S8. FTIR maps, generated at 700 cm^{-1} and 1660 cm^{-1}, showing the presence of the coating layer and that of the protein after HSA dead-end filtration through a membrane coated with PS_{100}-b-PAA_{107} (pH = 5 and 7; [HSA]= 1 mg·mL^{-1}).

As previously mentioned, the permeability measurements were performed before and after dead-end filtration. In fact, the result was around 36 L·m^{2}·h^{-1}·bar^{-1} (Figure S9) before HSA filtration, higher than that obtained earlier for membrane coated with the same copolymer and that yielded around 30 L·m^{2}·h^{-1}·bar^{-1}. Afterward, the flux evolution during HSA filtration was followed as shown in Figure S9, and it was found to decrease to 29 L·m^{2}·h^{-1}, after which a volume of 7 mL was passed through the membrane (*VRF* = 3.3).

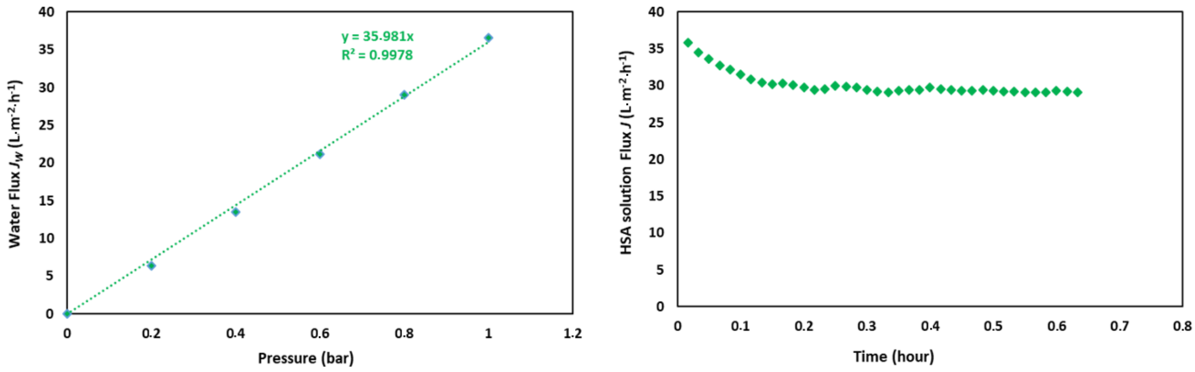

Figure S9. Plot of the water flux versus pressure from which the permeability was calculated for membranes coated with PS_{100}-b-PAA_{107}, and evolution of HSA solution flux versus time during HSA filtration (pH=5) through this same membrane.
